# Prevalence and In-hospital outcomes of diabetes among acute ischemic stroke patients in china: results from the Chinese Stroke Center Alliance

**DOI:** 10.1007/s00415-022-11112-z

**Published:** 2022-05-05

**Authors:** Guoliang Hu, Hongqiu Gu, Yingyu Jiang, Xin Yang, Chunjuan Wang, Yong Jiang, Zixiao Li, Yongjun Wang, Yilong Wang

**Affiliations:** 1 Department of Neurology, Beijing Tiantan Hospital, Capital Medical University, No 119 South 4th Ring West Road, Fengtai District, Beijing, 100070 China; 2 China National Clinical Research Center for Neurological Diseases, Beijing, China; 3 Advanced Innovation Center for Human Brain Protection, Capital Medical University, Beijing, China; 4 Beijing Key Laboratory of Translational Medicine for Cerebrovascular Disease, Beijing, China; 5 Chinese Institute for Brain Research, Beijing, China

**Keywords:** Acute ischemic stroke, Diabetes, Prevalence, In-hospital outcomes, Mortality, MACEs

## Abstract

**Background:**

Patients with ischemic stroke and diabetes are classified as extreme risk for secondary prevention, with much attention and specific management. However, the up-to-date information regarding the burden of diabetes in acute ischemic stroke (AIS) patients is lacking in China, and evidence for an association between diabetes and in-hospital outcomes after AIS remains controversial.

**Methods:**

This quality improvement study was conducted at 1,476 participating hospitals in the Chinese Stroke Center Alliance between 2015 and 2019. Prevalence of diabetes was evaluated in the overall study population and different subgroups. The association between diabetes and in-hospital outcomes in AIS patients was analyzed by using multivariable logistic regression analysis and propensity score-matched analysis.

**Results:**

Of 838,229 patients with AIS, 286,252 (34.2%) had diabetes/possible diabetes. The prevalence of diabetes/possible diabetes was higher in women than in men (37.6% versus 32.1%). Patients with diabetes/possible diabetes had higher rates of adverse in-hospital outcomes than those without. Multivariable analysis revealed a significant association between diabetes/possible diabetes and adverse in-hospital outcomes (all-cause mortality: odds ratio [OR], 1.30 [95% confidence interval [CI], 1.23–1.38]; major adverse cardiovascular events (MACEs): OR, 1.08 [95% CI, 1.06–1.10]) in AIS patients. The excess risk of in-hospital outcomes still remained in AIS patients with diabetes/possible diabetes after propensity score-matching analysis (all-cause mortality: OR, 1.26 [95% CI, 1.17–1.35]; MACEs: OR, 1.07 [95% CI, 1.05–1.10]).

**Conclusion:**

Diabetes was highly prevalent among AIS patients in China and associated with worse in-hospital outcomes. Greater efforts to increase targeted approach to secondary prevention treatments of diabetes in AIS patients are warranted.

**Supplementary Information:**

The online version contains supplementary material available at 10.1007/s00415-022-11112-z.

## Introduction

Stroke is the leading cause of death in China, accounting for approximately one-third of worldwide stroke-related death [1, 2]. Diabetes is a common comorbidity in stroke patients and is associated with adverse outcomes after stroke, particularly recurrent stroke and mortality [3–6]. Patients with ischemic stroke and diabetes are classified as extreme risk for secondary prevention, with much attention and specific management according to algorithmic guidelines published by diabetes, endocrinology, and stroke associations [7]. However, in China the up-to-date information regarding the burden of diabetes in acute ischemic stroke (AIS) patients is lacking. The latest studies focusing on the prevalence of diabetes among Chinese AIS patients were conducted over 10 years ago [3, 5]. With the alarming increase in the prevalence of diabetes among the general population in China, it is necessary to re-evaluate the burden of diabetes among Chinese AIS patients.

Despite diabetes is an independent risk factor for the development of AIS, the evidence for an association between diabetes and in-hospital outcomes after AIS still remains controversial [4, 8–11]. Some studies have suggested that diabetes is an independently predictive factor for poor in-hospital outcomes after ischemic stroke [4, 8], but several studies have not [9–11]. The differences of results in previous studies on the relationship between diabetes and in-hospital outcomes of AIS patients could possibly be explained by inconsistent definition of diabetes or in-hospital outcomes, different confounding variables adjusted in multivariable analysis model, and different length of in-hospital stay of AIS inpatients [4, 8–11]. It is essential to perform further studies for clearly elucidating this issue among AIS patients.

The objective of this study therefore was to provide an updated estimation of the burden of diabetes among AIS patients in China and to evaluate the association between diabetes and in-hospital outcomes of AIS patients, using data from the Chinese Stroke Center Alliance (CSCA) program.

## Materials and methods

### Study design

The CSCA, launched in 2015, is a large, nationwide, multicenter, voluntary, multifaceted intervention, and continuous quality improvement initiative. Details of the design and methodology of the CSCA project have been published [12]. In brief, the study included 756 tertiary hospitals and 720 secondary hospitals from 31 provinces, autonomous regions, or municipalities in China. Data were collected by trained hospital personnel via the web-based patient data collection and management tool (Medicine Innovation Research Center, Beijing, China). The China National Clinical Research Center for Neurological Diseases served as the data analysis center. Institutional review board approval was granted for this program with a waiver for informed consent by the Ethics Committee of Beijing Tiantan Hospital. The CSCA study was performed according to the principles expressed in the Declaration of Helsinki.

### Study population

The CSCA consecutively enrolled patients aged over 18 years having a primary diagnosis of acute stroke or transient ischemic attack (TIA) confirmed by brain computed tomography or magnetic resonance imaging, including ischemic stroke, TIA, intracerebral hemorrhage, or subarachnoid hemorrhage within 7 days of symptom onset. A total of 1,006,798 inpatient with stroke or TIA were registered between August 2015 and July 2019 from 1,476 hospitals. More details can be found in previous report [13]. Of these, 838,229 AIS inpatients were included in the current study after excluding patients with TIA, hemorrhagic stroke, subarachnoid hemorrhage, or diagnosed as "undetermined". The flow chart of study population recruitment is presented in Supplementary Fig. S1.

## Study variables

### Definition of diabetes

Diabetes was defined as a self-reported history of diabetes, or use of hypoglycemic medications before hospitalization or at discharge, or glycated hemoglobin A1c (HbA1c) ≥ 6.5% (48 mmol/mol), or diabetes listed in the medical records as one of the discharge diagnoses.

Possible diabetes was defined as elevated level of fasting blood glucose (FBG, ≥ 7.0 mmol/L (126 mg/dL)) but without measurement of HbA1c, considering we were unable to distinguish this group of patients between undiagnosed diabetes and stress hyperglycemia only based on the results of FBG.

### Definition of in‑hospital outcomes

The in-hospital outcomes investigated in this study referred to all-cause mortality and major adverse cardiovascular events (MACEs) during hospitalization. MACEs were defined as a combination of ischemic stroke, hemorrhagic stroke, TIA, and myocardial infarction (MI).

### Statistical analysis

Consider that most inpatients with possible diabetes who may be undiagnosed or at high risk of developing diabetes also require the same in-hospital care as inpatients with diabetes [14, 15], we combined diabetes and possible diabetes for statistical analysis for the purposes of our study. We first assessed the prevalence of diabetes/possible diabetes among all AIS patients as well as in different subgroups by sex. The baseline characteristics, in-hospital treatments, and in-hospital outcomes were compared between patients with and without diabetes/possible diabetes. Continuous variables were described as mean (standard deviation [SD]) for normally distributed data. Categorical variables were presented as the number (percentage). Patients' characteristics, in-hospital treatments, and in-hospital outcomes were compared using absolute standardized difference (ASD), with an ASD ≥ 10% considered clinically significant [16]. We used logistic regression analyses to evaluate the association between diabetes/possible diabetes and in-hospital outcomes, after adjusting for potential confounders, including baseline characteristics, risk factors, medical history, in-hospital treatments, and stroke severity.

To account for missing values on National Institutes of Health Stroke Scale (NIHSS) score, multiple imputation with chained equations was performed by using 10 iterations to impute missing values of NIHSS score, and the risk of diabetes/possible diabetes was recalculated in model 3 adjusting the imputed NIHSS score. Meanwhile, sensitivity analysis excluding patients with stress hyperglycemia (FBG ≥ 7.0 mmol/L (126 mg/dL) but with HbA1c < 6.5% (48 mmol/mol)) was also performed, considering the elevated risk of adverse in-hospital outcomes among those patients.

Subgroup analyses, including age (< 75/ ≥ 75 years old), sex (men/women), hypertension (yes/no), and in-hospital NIHSS score (0–4/5–14/ ≥ 15), were conducted using multivariable logistic regression model. The odds ratios (ORs) between the analyzed subgroups were compared by a Z-test.

Furthermore, propensity score-matched analysis was conducted to confirm the association between diabetes/possible diabetes and in-hospital outcomes. We calculated propensity scores of having diabetes/possible diabetes by logistic regression analysis involving the following factors: age, sex, body mass index (BMI), hypertension, dyslipidemia, current smoking, drinking, history of stroke/TIA, history of MI, history of atrial fibrillation, history of heart failure, history of carotid stenosis, history of peripheral artery disease, history of liver/renal dysfunction, in-hospital NIHSS score, insurance and hospital location. Patients with and without diabetes/possible diabetes were then paired at 1:1 according to the propensity scores using nearest-neighbor matching without replacement, with a caliper size of 0.02. The ASDs of variables were compared before and after propensity score matching (Supplementary Fig. S2), with ASD < 10% for the variables being considered successful balancing between groups. The baseline characteristics and in-hospital treatments between groups were re-compared after propensity score matching.

Statistical analyses were performed using SAS 9.4 (SAS Institute, Cary, NC, USA). Two-tailed P values of < 0.05 were considered statistically significant. Descriptive tables were produced by an SAS macro (%ggBaseline) that can automatically generate baseline tables [17].

## Results

### Diabetes prevalence in AIS patients

Among 838,229 patients with AIS who were included in this study, 524,351 (62.6%) were men and 313,878 (37.4%) were women, with an average age of 66.2 (± 12.0) years. Of these patients, a total of 286,252 (34.2%) had diabetes/possible diabetes (Table 1), including 33.4% diabetes and 0.7% possible diabetes. Female patients had a higher prevalence of diabetes/possible diabetes than male patients (37.6% versus 32.1%). Diabetes/possible diabetes was most common among patients aged 55–64 years (men: 34.6%; women: 41.6%). The prevalence of diabetes/possible diabetes increased with BMI. Patients with a history of stroke/TIA had a higher proportion of diabetes/possible diabetes than those without history of stroke/TIA (38.2% versus 32.3%).

**Table 1:** Prevalence of diabetes/possible diabetes in AIS patients

	Total AIS (*N* = 838,229)	Men (*N* = 524,351)	Women (*N* = 313,878)
Total, *n* (% [95% CI])	286,252 (34.2 [34.1–34.3])	168,152 (32.1 [31.9–32.2])	118,100 (37.6 [37.5–37.8])
Age, *n* (% [95% CI]) (years)			
< 45	7,711 (24.3 [23.8–24.8])	6,131 (24.4 [23.9–25.0])	1,580 (23.7 [22.7–24.7])
45–54	38,864 (32.2 [31.9–32.5])	27,744 (31.8 [31.5–32.1])	11,120 (33.1 [32.6–33.6])
55–64	76,844 (36.9 [36.7–37.1])	48,570 (34.6 [34.4–34.9])	28,274 (41.6 [41.3–42.0])
65–74	92,979 (36.5 [36.3–36.7])	50,873 (33.2 [33.0–33.5])	42,106 (41.4 [41.1–41.7])
≥ 75	69,854 (31.4 [31.2–31.5])	34,834 (29.3 [29.1–29.6])	35,020 (33.7 [33.4–33.9])
BMI^a^, *n* (% [95% CI])			
< 24.0	138,337 (30.9 [30.8–31.0])	79,606 (29.0 [28.8–29.1])	58,731 (33.9 [33.7–34.2])
24.0 ≤ BMI < 28.0	112,240 (36.6 [36.5–36.8])	69,949 (34.5 [34.3–34.7])	42,291 (40.8 [40.5–41.1])
≥ 28.0	30,677 (43.6 [43.3–44.0])	15,734 (40.9 [40.4–41.4])	14,943 (46.9 [46.3–47.4])
Prior stroke/TIA, *n* (% [95% CI])			
Yes	100,603 (38.2 [38.0–38.4])	60,002 (35.8 [35.5–36.0])	40,601 (42.4 [42.1–42.7])
No	185,649 (32.3 [32.2–32.4])	108,150 (30.3 [30.2–30.5])	77,499 (35.5 [35.3–35.7])

*AIS* acute ischemic stroke, *BMI* body mass index, *TIA* transient ischemic attack

^a^BMI, data of BMI were not available for 13,629 patients

### Characteristics of AIS patients with/without diabetes

The demographics and clinical characteristics of included patients are summarized in Table 2. Compared with AIS patients without diabetes, patients with diabetes/possible diabetes were more likely to have an increased BMI (24.5 ± 5.0 versus 23.7 ± 4.0, ASD = 17.7) and a higher proportion of hypertension (91.0% versus 85.7%, ASD = 16.6), dyslipidemia (18.8% versus 11.7%, ASD = 19.8), and a history of stroke/TIA (35.1% versus 29.5%, ASD = 12.0). The severity of stroke (expressed as NIHSS score) showed similar results in AIS patients with and without diabetes/possible diabetes.

**Table 2:** Clinical characteristics of AIS patients with or without diabetes/possible diabetes

	Total (*N* = 838,229)	Diabetes/possible diabetes (*N* = 286,252)	No diabetes (*N* = 551,977)	ASD (%)
Age, mean (SD), years	66.2 (12.0)	66.2 (11.2)	66.2 (12.4)	0.0
Women, *n* (%)	313,878 (37.4)	118,100 (41.3)	195,778 (35.5)	11.9
BMI^a^, mean (SD)	24.0 (4.4)	24.5 (5.0)	23.7 (4.0)	17.7
BMI^a^, *n* (%)				
< 24.0	447,945 (54.3)	138,337 (49.2)	309,608 (57.0)	15.7
24.0 ≤ BMI < 28.0	306,331 (37.1)	112,240 (39.9)	194,091 (35.7)	8.7
≥ 28.0	70,324 (8.5)	30,677 (10.9)	39,647 (7.3)	12.5
Education, *n* (%)				
College	24,727 (2.9)	9,539 (3.3)	15,188 (2.8)	2.9
High school	249,783 (29.8)	89,015 (31.1)	160,768 (29.1)	4.4
Below elementary	253,266 (30.2)	81,647 (28.5)	171,619 (31.1)	5.7
Unclear	310,453 (37.0)	106,051 (37.0)	204,402 (37.0)	0.0
Insurance, *n* (%)				
UEBMI	240,261 (28.7)	96,103 (33.6)	144,158 (26.1)	16.4
URBMI	158,383 (18.9)	55,134 (19.3)	103,249 (18.7)	1.5
NRCMS	351,637 (41.9)	104,845 (36.6)	246,792 (44.7)	16.5
Self-pay	53,365 (6.4)	17,581 (6.1)	35,784 (6.5)	1.6
Other	34,583 (4.1)	12,589 (4.4)	21,994 (4.0)	2.0
SBP^b^, mean (SD)	150.0 (23.1)	151.2 (23.0)	149.4 (23.1)	7.8
DBP^c^, mean (SD)	87.0 (13.9)	86.7 (13.7)	87.1 (14.0)	2.9
Risk factors				
Hypertension, *n* (%)	733,705 (87.5)	260,522 (91.0)	473,183 (85.7)	16.6
Dyslipidemia, *n* (%)	118,187 (14.1)	53,736 (18.8)	64,451 (11.7)	19.8
Current smoking, *n* (%)				
Men	191,153 (36.5)	56,696 (33.7)	134,457 (37.7)	8.4
Women	8,614 (2.7)	2,792 (2.4)	5,822 (3.0)	3.7
Drinking, *n* (%)	196,131 (23.4)	62,684 (21.9)	133,447 (24.2)	5.5
History of diseases				
Prior stroke/TIA, *n* (%)	263,509 (31.4)	100,603 (35.1)	162,906 (29.5)	12.0
Prior MI, *n* (%)	14,378 (1.7)	6,411 (2.2)	7,967 (1.4)	6.0
Atrial fibrillation, *n* (%)	44,802 (5.3)	14,319 (5.0)	30,483 (5.5)	2.2
Heart failure, *n* (%)	8,978 (1.1)	3,429 (1.2)	5,549 (1.0)	1.9
Carotid stenosis, *n* (%)	10,929 (1.3)	4,542 (1.6)	6,387 (1.2)	3.4
PAD, *n* (%)	14,648 (1.7)	7,047 (2.5)	7,601 (1.4)	8.0
Liver/renal dysfunction, *n* (%)	8,447 (1.0)	4,070 (1.4)	4,377 (0.8)	5.8
NIHSS^d^, *n* (%)				
0–4	429,564 (64.3)	143,004 (63.0)	286,560 (65.0)	4.2
5–14	196,594 (29.4)	69,207 (30.5)	127,387 (28.9)	3.5
≥ 15	42,071 (6.3)	14,881 (6.6)	27,190 (6.2)	1.6
Hospital location, *n* (%)				
Eastern	385,913 (46.0)	137,284 (48.0)	248,629 (45.0)	6.0
Central	277,269 (33.1)	89,981 (31.4)	187,288 (33.9)	5.3
Western	175,047 (20.9)	58,987 (20.6)	116,060 (21.0)	1.0

*AIS *acute ischemic stroke,* ASD *absolute standard difference,* BMI *body mass index,* UEBMI *urban employee basic medical insurance,* URBMI *urban resident basic medical insurance,* NRCMS *new rural cooperative medical scheme,* SBP *systolic blood pressure,* DBP *diastolic blood pressure,* TIA *transient ischemic attack,* MI *myocardial infarction,* PAD *peripheral artery disease* NIHSS *national institutes of health stroke scale

^a^BMI, data of BMI were not available for 13,629 patients

^b^SBP, data of SBP were not available for 285 patients

^c^DBP, data of DBP were not available for 291 patients

^d^NIHSS, data of NIHSS were not available for 170,000 patients

### In‑hospital treatments of AIS patients with/without diabetes

The in-hospital treatments were compared between AIS patients with and without diabetes/possible diabetes (Table 3). There were no significant differences between two groups among in-hospital treatments, including IV-rtPA administration ≤ 4.5 h, antiplatelet therapy, anticoagulant therapy, and statins. Longer length of hospital stay was observed in patients with diabetes/possible diabetes than those without diabetes in this study (12.1 ± 7.1 versus 11.4 ± 6.6, ASD = 10.2).

**Table 3:** In-hospital treatments of AIS patients with or without diabetes/possible diabetes

	Total (*N* = 838,229)	Diabetes/possible diabetes (*N* = 286,252)	No diabetes (*N* = 551,977)	ASD (%)
IV-rtPA administration ≤ 4.5 h, *n* (%)	44,978 (5.5)	13,597 (4.8)	31,381 (5.8)	4.5
Antiplatelet drugs, *n* (%)	689,436 (83.8)	235,265 (83.8)	454,171 (83.9)	0.3
Aspirin	609,170 (74.1)	206,899 (73.7)	402,271 (74.3)	1.4
Clopidogrel	391,507 (47.6)	137,523 (49.0)	253,984 (46.9)	4.2
Aspirin + clopidogrel	316,733 (38.5)	111,014 (39.5)	205,719 (38.0)	3.1
Anticoagulant, *n* (%)	45,060 (5.5)	14,992 (5.3)	30,068 (5.6)	1.3
Unfractionated heparin	3,866 (0.5)	1,360 (0.5)	2,506 (0.5)	0.0
Low molecular weight heparin	32,319 (3.9)	10,931 (3.9)	21,388 (3.9)	0.0
Warfarin	9,517 (1.2)	2,781 (1.0)	6,736 (1.2)	1.9
Other anticoagulants	4,143 (0.5)	1,379 (0.5)	2,764 (0.5)	0.0
Statins, *n* (%)	51,791 (6.2)	18,422 (6.4)	33,369 (6.0)	1.7
Length of stay^a^, mean (SD), days	11.6 (6.7)	12.1 (7.1)	11.4 (6.6)	10.2

*AIS* acute ischemic stroke, *ASD* absolute standard difference, *IV-rtPA* intravenous recombinant tissue plasminogen activator

^a^ Length of stay, data of length of stay were not available for 4,369 patients

### Association between diabetes and in‑hospital outcomes

We compared the in-hospital outcomes between AIS patients with and without diabetes/possible diabetes (Supplementary Table S1). Higher rates of in-hospital outcomes were observed in AIS patients with diabetes/possible diabetes than those without diabetes, including all-cause mortality (0.8% versus 0.5%) and MACEs (7.7% versus 6.1%). In univariate logistic regression analysis, we observed significantly higher risk of in-hospital all-cause mortality and MACEs in AIS patients with diabetes/possible diabetes (Table 4, Supplementary Table S2). After adjusting for patients' demographic characteristics, medical history, in-hospital treatments, and other confounding factors, diabetes/possible diabetes was associated with excess risk of in-hospital all-cause mortality (odds ratio [OR], 1.38 [95% CI 1.30–1.46]) and MACEs (OR, 1.09 [95% CI 1.07–1.12]). After further adjusting in-hospital NIHSS score, the higher risk of in-hospital all-cause mortality (OR, 1.33 [95% CI 1.24–1.42]) and MACEs (OR, 1.08 [95% CI 1.06–1.10]) were still statistically significant in patients with diabetes/possible diabetes. Moreover, after multiple imputation with chained equations being used to impute missing value of NIHSS score, the significant association between diabetes/possible diabetes and in-hospital outcomes (all-cause mortality: OR, 1.30 [95% CI 1.23–1.38]; MACE: OR, 1.08 [95% CI 1.06–1.10]) still remained in AIS patients after multivariable adjustment (Table 4).

**Table 4:** Association between diabetes/possible diabetes and in-hospital outcomes in AIS patients

	Unadjusted		Adjusted: Model 1		Adjusted: Model 2		Adjusted: Model 3	
	Unadjusted OR (95% CI)	*p* value	Adjusted OR (95% CI)	*p* value	Adjusted OR (95% CI)	*p* value	Adjusted OR (95% CI)	*p* value
All-cause mortality	1.48 (1.40–1.56)	< 0.001	1.38 (1.30–1.46)	< 0.001	1.33 (1.24–1.42)	< 0.001	1.30 (1.23–1.38)	< 0.001
MACEs	1.27 (1.24–1.29)	< 0.001	1.09 (1.07–1.12)	< 0.001	1.08 (1.06–1.10)	< 0.001	1.08 (1.06–1.10)	< 0.001
Recurrent ischemic stroke	1.34 (1.31–1.37)	< 0.001	1.13 (1.11–1.15)	< 0.001	1.12 (1.10–1.15)	< 0.001	1.12 (1.10–1.15)	< 0.001
Recurrent hemorrhagic stroke	1.08 (1.03–1.13)	0.001	1.07 (1.02–1.13)	0.005	1.03 (0.98–1.09)	0.297	1.03 (0.98–1.08)	0.211
TIA	1.10 (1.05–1.16)	< 0.001	0.95 (0.90–1.00)	0.056	0.91 (0.86–0.97)	0.004	0.95 (0.90–1.01)	0.078
MI	1.36 (1.27–1.46)	< 0.001	1.11 (1.03–1.20)	0.005	1.08 (1.00–1.18)	0.065	1.09 (1.01–1.18)	0.020

*AIS* acute ischemic stroke, *OR* odds ratio, *CI* confidence interval, *MACEs* major adverse cardiovascular events, *TIA* transient ischemic attack, *MI* myocardial infarction

Model 1: age, sex, body mass index, hypertension, dyslipidemia, current smoking, drinking, history of stroke/TIA, history of carotid stenosis, history of MI, history of atrial fibrillation, history of heart failure, history of PAD, history of liver/renal dysfunction, administration of antiplatelet agent use, administration of anticoagulant therapy and administration of statins, education, insurance and hospital location

Model 2: adjusted for variables in model 1 plus in-hospital National Institutes of Health Stroke Scale score

Model 3: adjusted for variables in model 1 plus imputed in-hospital National Institutes of Health Stroke Scale score

Sensitivity analysis was also performed to evaluate the association between diabetes/possible diabetes and in-hospital outcomes (Supplementary Table S3, Supplementary Table S4). After excluding patients with possible stress hyperglycemia (*n* = 33,706) in patients without diabetes, diabetes/possible diabetes was still significant associated with higher risk of in-hospital outcomes, including all-cause mortality (OR, 1.48 [95% CI 1.39–1.58]) and MACEs (OR, 1.09 [95% CI 1.07–1.11]) (Supplementary Table S3). Even excluding in-hospital TIA from MACEs, the higher risk of MACEs still remained in patients with diabetes/possible diabetes than those without diabetes (Supplementary Table S4).

**Fig. 1:**
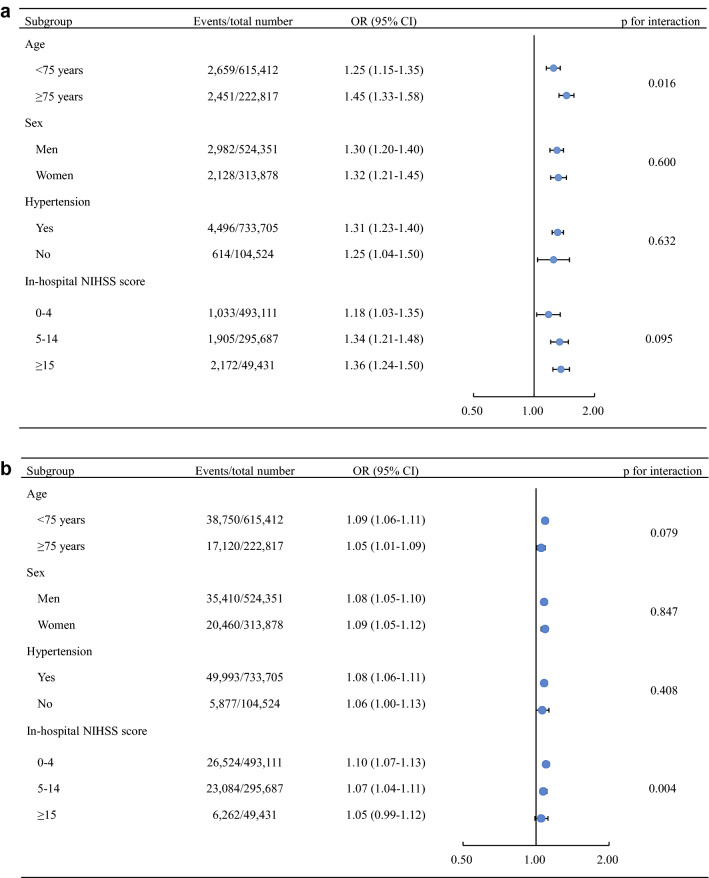
Subgroup analysis for the association between diabetes/possible diabetes and in-hospital outcomes in AIS patients. **a** Association between diabetes/possible diabetes and in-hospital all-cause mortality. **b** Association between diabetes/possible diabetes and in-hospital MACEs. *OR* odds ratio, *CI* confidence interval, *MACEs* major adverse cardiovascular events, *NIHSS* national institutes of health stroke scale

In further subgroup analyses using multivariable logistic regression, higher risks of in-hospital all-cause mortality and MACEs were observed in patients with diabetes/possible diabetes in all subgroups (Fig. 1a,b).

### Propensity score-matching analysis

In propensity score matching, 221,431 AIS patients with diabetes/possible diabetes were matched with 221,431 patients without diabetes. After matching, the ASD were all less than 10.0% for the variables included in the calculation of propensity score, suggesting that AIS patients with and without diabetes/possible diabetes were well matched (Supplementary Fig. S2). The baseline characteristics and in-hospital treatments between groups were re-compared (Supplementary Table S5). The rates of in-hospital outcomes still remained higher in patients with diabetes/possible diabetes than those without diabetes (all-cause mortality: 0.8% versus 0.6%; MACEs: 7.4% versus 6.9%) (Supplementary Table S1), and patients with diabetes/possible diabetes had higher risks of in-hospital all-cause mortality (OR, 1.26 [95% CI 1.17–1.35]) and MACEs (OR, 1.07 [95% CI 1.05–1.10]) than those without diabetes (Supplementary Table S6). After excluding in-hospital TIA from MACEs, the higher risk of MACEs still remained in diabetic/possible diabetic patients than in non-diabetic patients (Supplementary Table S4).

## Discussion

To the best of our knowledge, the present study is the largest contemporary registry study to provide an updated estimation of the burden of diabetes among AIS patients in China and evaluate the association between diabetes and in-hospital outcomes after AIS. Our findings are of great significance in pointing out the future direction for improving secondary prevention strategies of AIS in China.

### High diabetes prevalence among AIS patients in China

In this study, we found that diabetes/possible diabetes was highly prevalent in AIS patients across China, with 1 in 3 AIS patients had diabetes/possible diabetes.

The prevalence of diabetes in our study is higher than that in previous reports [5, 18–20], which may reflect the increasing prevalence of diabetes among AIS patients in China. The China National Stroke Registry reported that 21.6% of AIS patients across China had diabetes from 2007–2008, and 20.7% of them from 2012–2013 [18]. Date from the China National Stroke Screening Survey from 2013 to 2015 showed that 21.5% of ischemic stroke patients had diabetes across China [19]. Moreover, in view of the alarming growth in the prevalence of diabetes among the general population in China [21, 22], the proportion of diabetes among Chinese AIS patients will be continuously increasing.

Furthermore, according to the Survey on Abnormal Glucose Regulation in Patients With Acute Stroke Across China (ACROSS-China) from 2008 to 2009, 23% of patients with AIS were diagnosed with diabetes by medical history, and notably 22.8% of these patients were newly diagnosed with diabetes by oral glucose tolerance test (OGTT) [3]. In this setting, the actual prevalence of diabetes in AIS patients may be even higher than the reported proportion of our study, as some patients with diabetes may not have been identified because in the routine clinical practice OGTT is not widely applied to detect diabetes among inpatients. The finding from ACROSS-China also showed that the proportion of impaired glucose regulation in AIS patients was 23.9%, indicating that about a quarter of AIS patients in China are in pre-diabetes [3]. These findings suggest that neurologists in China have to manage a large proportion of AIS patients with pre-diabetes/diabetes in their clinical practice. Therefore, the screening for diabetes should be included in the routine examination at admission as the first key step in neurologists' management strategies. Clinical guidelines have suggested that it was reasonable for all patients with stroke to have an assessment of diabetes at admission [14, 23]. It is time to attach importance to the professional training of neurologists toward diabetes education and care and strengthen multi-disciplinary cooperation between internists, endocrinologists, and neurologists in clinical practice.

### Adverse impact of diabetes on in-hospital outcomes of AIS patients

Our study provided evidence of an association between diabetes and adverse in-hospital outcomes in AIS patients. We found that AIS patients with diabetes/possible diabetes had higher risks of in-hospital all-cause mortality and MACEs, especially stroke recurrence, than did without diabetes, in both multivariable logistic regression analysis and propensity-score matching analysis.

Consistent with previous studies [4, 5, 24], we observed higher crude rates of adverse in-hospital outcomes in AIS patients with diabetes/possible diabetes than those without diabetes. Notably, the rate of in-hospital adverse outcomes was lower in our study than that in previous studies conducted among Chinese AIS patients [5, 25]. These findings might indicate that the advancements in the management of ischemic stroke patients during the past two decades have improved the prognosis of patients with ischemic stroke but have not eliminated the risk gap between diabetic and non-diabetic patients. Similar to those in previous studies [4, 5], AIS patients with diabetes in the present study were more likely to be overweight, had a higher proportion of history of stroke/TIA and more comorbidities, including hypertension and dyslipidemia, than those without diabetes. In our study, in-hospital treatments for AIS were comparable between patients with and without diabetes. After adjusting for confounding variables, the excess risk of adverse in-hospital outcomes still remained in AIS patients with diabetes/possible diabetes, which was in line with previous findings [4, 8]. Therefore, all AIS patients with diabetes/possible diabetes should raise much attention in clinical practice. In particular, female AIS patients should be kept under special close supervision in light of their high prevalence of diabetes and high risk of poor prognosis [26]. However, there has been some studies regarding the association between diabetes and in-hospital outcomes after AIS showed that the adverse in-hospital outcomes after ischemic stroke were not increased in patients with diabetes [9–11]. Many factors could contribute to these differences, including type II error result from smaller samples of study, different treatment patterns between countries, ethnicity difference, population health, and other social determinants.

Moreover, patients with diabetes/possible diabetes were more likely to have a slightly longer hospital stay. These observations fit with previous studies that showed that diabetes resulted in longer length of stay which possibly meant the complex complications, severe clinical conditions, and difficulties in controlling the blood glucose during hospitalization [8, 24]. Also, some studies with longer follow-up periods in Chinese population have found significant associations between diabetes and poor clinical prognosis among AIS patients [5, 27]. Those findings highlight that early identification and treatment of diabetes is more than timely and much needed for improving the prognosis of AIS patients. Previous studies showed that the presence of a prior TIA was associated with a favorable outcome in ischemic stroke patients, suggesting a neuroprotective effect of TIA possibly by inducing a phenomenon of ischemic tolerance allowing better recovery from a subsequent ischemic stroke [28, 29]. However, some studies failed to confirm this [30]. The discrepancy of results may result from the difference in the type of stroke, inconsistent definition of study endpoint, and bias in the record of previous TIA. Future prospective studies with carefully design are need to explore the phenomenon of ischemic tolerance in AIS patients.

Nowadays, clinical guidelines have classified stroke patients with diabetes as a high-risk group, and management strategies for them have been specially issued by the national and international guidelines [7, 14, 31–33]. With much attention being paid to those patients, the heavy burden and excess risk of diabetes in AIS patients are expected to decrease in the near future.

## Limitations

Several limitations need to be addressed. First, the data on OGTT were unavailable in our study, which might influence the evaluation of proportion of AIS patients with diabetes. However, OGTT was not routinely applied in the current clinical workup. Further studies that consider OGTT are warranted. Second, as this was a quality improvement study based on medical records, the information about physical activity, diabetes types and emerging cerebrovascular risk factors (e.g., sleep-related breathing disorders, drug abuse, oral contraceptive use and inflammatory markers) were not available, which future studies should take into consideration. Third, this study did not collect information on the specific time of FBG measurement during hospitalization, some patients with only elevated FBG could not be definitively diagnosed with diabetes. Nevertheless, the use of only FBG test indicated that neurologists do not pay enough attention to the diagnosis of diabetes in AIS patients. Fourth, the potential bias in diagnostic criteria between participating hospitals may underestimate the endpoints. However, we still found the adverse effects of diabetes on in-hospital outcomes for AIS patients. Finally, the CSCA is based on voluntary enrollment of hospitals and does not have an elaborately designed sampling mechanism. However, the large sample size improves the robustness and generalizability of this study.

## Conclusions

Our results showed that diabetes was highly prevalent among AIS patients in China and independently associated with worse in-hospital outcomes, based on a nationwide representative registry study. These findings highlight the importance and necessity of early identification and timely management of diabetes in AIS patients, informing future priorities for improving secondary prevention of AIS patients in clinical practice in China.

## Supplementary Information

Below is the link to the electronic supplementary material.

Supplementary file1 (DOCX 10121 KB)

## Data Availability

The datasets analyzed during the current study are not publicly available due to intellectual property rights, but are available from the corresponding author on reasonable request.

## Abbreviations

AIS: Acute ischemic stroke
ASD: Absolute standardized difference
BMI: Body mass index
CI: Confidence interval
DBP: Diastolic blood pressure
HbA1c: Glycated hemoglobin A1c
IQR: Interquartile range
IV rtPA: Intravenous tissue plasminogen activator
MACEs: Major adverse cardiovascular events
MI: Myocardial infarction
NIHSS: National Institutes of Health Stroke Scale
OGTT: Oral glucose tolerance test
OR: Odds ratio
SBP: Systolic blood pressure
SD: Standard deviation
TIA: Transient ischemic attack
