# Supplementary material for: Prevalence and In-hospital outcomes of diabetes among acute ischemic stroke patients in china: results from the Chinese Stroke Center Alliance
Source: J Neurol. 2022 May 5;269(9):4772–82. doi: 10.1007/s00415-022-11112-z (PMC9363385; doi:10.1007/s00415-022-11112-z)
Supplement: Supplementary file 1 — Supplementary file1 (DOCX 10121 KB) [file 415_2022_11112_MOESM1_ESM.docx]

**SUPPLEMENTARY MATERIAL**

**Prevalence and In-Hospital Outcomes of Diabetes Among Acute Ischemic Stroke Patients in China: Results from the Chinese Stroke Center Alliance**

Guoliang Hu, PhD ^a,b,c,d^; Hongqiu Gu, PhD ^b^; Yingyu Jiang, MHS ^b^; Xin Yang, MD ^b^; Chunjuan Wang, MD ^b^; Yong Jiang, PhD ^b^; Zixiao Li, MD ^a,b,e^; Yongjun Wang, MD, PhD ^a,b,c,d^; Yilong Wang, MD, PhD ^a,b,c,d,e^

^a^ Department of Neurology, Beijing Tiantan Hospital, Capital Medical University, Beijing, China

^b^ China National Clinical Research Center for Neurological Diseases, Beijing, China

^c^ Advanced Innovation Center for Human Brain Protection, Capital Medical University, Beijing, China

^d^ Beijing Key Laboratory of Translational Medicine for Cerebrovascular Disease, Beijing, China

^e^ Chinese Institute for Brain Research, Beijing, China

**Corresponding Author:**

Yilong Wang

Department of Neurology, Beijing Tiantan Hospital, Capital Medical University,

No 119 South 4th Ring West Road, Fengtai District, Beijing 100070, China

E-mail: yilong528@gmail.com

[Supplementary Methods 3](#_Toc98971966)

[Table S1 Comparison of in-hospital outcomes in AIS patients with and without diabetes/possible diabetes 4](#_Toc98971967)

[Table S2 Univariate analysis of the association between diabetes/possible diabetes and in-hospital outcomes 5](#_Toc98971968)

[Table S3 Sensitivity analysis for the association between diabetes/possible diabetes and in-hospital outcomes in AIS patients 7](#_Toc98971969)

[Table S4 Sensitivity analysis for the association between diabetes/possible diabetes and MACEs in AIS patients 8](#_Toc98971970)

[Table S5 Characteristics of AIS patients with and without diabetes/possible diabetes after propensity-score matching 9](#_Toc98971971)

[Table S6 Association between diabetes/possible diabetes and in-hospital outcomes in AIS patients after propensity-score matching 13](#_Toc98971972)

[Fig. S1 Flow chart of study 14](#_Toc98971973)

[Fig. S2 Love plot for absolute standardized differences before and after propensity score matching 15](#_Toc98971974)

# Supplementary Methods

**Study variables**

Patients with hypertension were defined as those with a history of hypertension, or receiving antihypertensive therapy before hospitalization or at discharge, or systolic blood pressure ≥ 140 mmHg or diastolic blood pressure ≥ 90 mmHg at admission, or hypertension listed in the medical records as one of the discharge diagnoses. Patients with dyslipidemia were defined as having a history of dyslipidemia, or lipid-lowering drug use before hospitalization. Patient history of other conditions, including stroke/transient ischemic attack, myocardial infarction, atrial fibrillation, heart failure, peripheral artery disease and liver/renal dysfunction was defined according to notes obtained from patients' original medical records. Body mass index (BMI) was defined as weight in kilograms divided by the square of height in metres. Low weight was defined as BMI<18.5, normal weight was defined as 18.5≤BMI<24.0, overweight was defined as 24.0≤BMI<28.0 and obesity was defined as BMI ≥28.0 kg/m^2^, according to the recommendations of the Working Group on Obesity in China [1]. The severity of stroke was scaled by the National Institutes of Health Stroke Scale score.

# Table S1 Comparison of in-hospital outcomes in AIS patients with and without diabetes/possible diabetes

|  | **The whole study population** | | | **Propensity score-matched population** | | |
| --- | --- | --- | --- | --- | --- | --- |
|  | Diabetes/possible diabetes  (N=286,252) | No diabetes  (N=551,977) | ASD (%) | Diabetes/possible diabetes  (N=221,431) | No diabetes  (N=221,431) | ASD (%) |
| All-cause mortality | 2,215 (0.8) | 2,895 (0.5) | 3.7 | 1,678 (0.8) | 1,334 (0.6) | 2.4 |
| MACEs | 21,931 (7.7) | 33,939 (6.1) | 6.3 | 16,433 (7.4) | 15,374 (6.9) | 1.9 |
| Recurrent ischemic stroke | 16,998 (5.9) | 24,874 (4.5) | 6.3 | 12,653 (5.7) | 11,331 (5.1) | 2.7 |
| Recurrent hemorrhagic stroke | 2,770 (1.0) | 4,947 (0.9) | 1.0 | 2,200 (1.0) | 2,197 (1.0) | 0.0 |
| TIA | 2,236 (0.8) | 3,919 (0.7) | 1.2 | 1,609 (0.7) | 1,742 (0.8) | 1.2 |
| MI | 1,425 (0.5) | 2,022 (0.4) | 1.5 | 1,051 (0.5) | 978 (0.4) | 1.5 |

AIS, acute ischemic stroke; ASD, absolute standard difference; MACEs: major adverse cardiovascular events; TIA: transient ischemic attack; MI: myocardial infarction

# Table S2 Univariate analysis of the association between diabetes/possible diabetes and in-hospital outcomes

|  | All-cause mortality | | MACEs | |
| --- | --- | --- | --- | --- |
|  | Unadjusted OR  (95% CI) | p value | Unadjusted OR  (95% CI) | p value |
| Diabetes/possible diabetes | 1.48 (1.40-1.56) | <0.001 | 1.27 (1.24-1.29) | <0.001 |
| Age | 1.04 (1.04-1.05) | <0.001 | 1.01 (1.01-1.01) | <0.001 |
| Women | 1.19 (1.13-1.26) | <0.001 | 0.96 (0.95-0.98) | <0.001 |
| BMI | 0.98 (0.97-0.99) | <0.001 | 1.01 (1.00-1.01) | <0.001 |
| Hypertension | 1.04 (0.96-1.14) | 0.332 | 1.23 (1.19-1.26) | <0.001 |
| Dyslipidemia | 1.29 (1.20-1.39) | <0.001 | 2.50 (2.45-2.55) | <0.001 |
| Current smoking | 0.70 (0.65-0.75) | <0.001 | 0.83 (0.81-0.85) | <0.001 |
| Drinking | 0.85 (0.79-0.91) | <0.001 | 0.99 (0.97-1.01) | 0.319 |
| Prior stroke/TIA | 1.51 (1.43-1.60) | <0.001 | 5.96 (5.85-6.08) | <0.001 |
| Carotid stenosis | 1.48 (1.21-1.82) | <0.001 | 3.00 (2.85-3.15) | <0.001 |
| Prior MI | 3.36 (2.97-3.79) | <0.001 | 3.31 (3.17-3.46) | <0.001 |
| Atrial fibrillation | 4.50 (4.20-4.82) | <0.001 | 2.04 (1.98-2.10) | <0.001 |
| Heart failure | 5.39 (4.76-6.10) | <0.001 | 2.70 (2.55-2.86) | <0.001 |
| PAD | 2.15 (1.86-2.49) | <0.001 | 2.82 (2.69-2.95) | <0.001 |
| Liver/renal dysfunction | 3.30 (2.82-3.87) | <0.001 | 1.98 (1.86-2.12) | <0.001 |
| Antiplatelet drugs | 0.34 (0.32-0.35) | <0.001 | 0.64 (0.63-0.65) | <0.001 |
| Anticoagulant therapy | 1.53 (1.38-1.69) | <0.001 | 1.63 (1.58-1.69) | <0.001 |
| Statins | 1.16 (1.04-1.29) | 0.006 | 0.94 (0.91-0.98) | 0.001 |
| Education |  |  |  |  |
| College vs. Below Elementary | 1.65 (1.43-1.91) | <0.001 | 1.11 (1.05-1.17) | <0.001 |
| High school vs. Below Elementary | 1.22 (1.13-1.31) | <0.001 | 1.08 (1.06-1.10) | <0.001 |
| Unclear vs. Below Elementary | 1.25 (1.17-1.34) | <0.001 | 1.00 (0.98-1.02) | 0.757 |
| Insurance |  |  |  |  |
| NRCMS vs. Self-pay | 0.52 (0.46-0.59) | <0.001 | 1.20 (1.15-1.25) | <0.001 |
| Other vs. Self-pay | 1.35 (1.16-1.58) | <0.001 | 1.38 (1.30-1.46) | <0.001 |
| UEBMI vs. Self-pay | 1.50 (1.34-1.68) | <0.001 | 1.47 (1.41-1.53) | <0.001 |
| URBMI vs. Self-pay | 0.92 (0.81-1.04) | 0.169 | 1.28 (1.23-1.34) | <0.001 |
| Hospital location |  |  |  |  |
| Eastern vs. Western | 0.68 (0.63-0.72) | <0.001 | 1.13 (1.10-1.16) | <0.001 |
| Central vs. Western | 0.66 (0.61-0.71) | <0.001 | 1.14 (1.11-1.16) | <0.001 |
| NIHSS | 1.13 (1.13-1.13) | <0.001 | 1.05 (1.05-1.05) | <0.001 |

AIS, acute ischemic stroke; BMI, body mass index; CI, confidence interval; MACEs: major adverse cardiovascular events; MI: myocardial infarction; NIHSS, national institutes of health stroke scale; NRCMS, new rural cooperative medical scheme; OR, odds ratio; PAD, peripheral artery disease; TIA: transient ischemic attack; UEBMI, urban employee basic medical insurance; URBMI, urban resident basic medical insurance

# Table S3 Sensitivity analysis for the association between diabetes/possible diabetes and in-hospital outcomes in AIS patients

|  | **Unadjusted OR (95% CI)** | **p value** | **Adjusted OR (95% CI)** | **p value** |
| --- | --- | --- | --- | --- |
| All-cause mortality | 1.76 (1.66-1.87) | <0.001 | 1.48 (1.39-1.58) | <0.001 |
| MACE | 1.30 (1.27-1.32) | <0.001 | 1.09 (1.07-1.11) | <0.001 |
| Recurrent ischemic stroke | 1.35 (1.32-1.38) | <0.001 | 1.13 (1.10-1.15) | <0.001 |
| Recurrent hemorrhagic stroke | 1.19 (1.13-1.25) | <0.001 | 1.08 (1.03-1.14) | 0.002 |
| TIA | 1.10 (1.04-1.16) | <0.001 | 0.94 (0.89-1.00) | 0.040 |
| MI | 1.46 (1.36-1.57) | <0.001 | 1.14 (1.06-1.23) | <0.001 |

AIS, acute ischemic stroke; OR, odds ratio; CI, confidence interval; MACEs: major adverse cardiovascular events; TIA: transient ischemic attack; MI: myocardial infarction.

# Table S4 Sensitivity analysis for the association between diabetes/possible diabetes and MACEs in AIS patients

|  | **Unadjusted** | | **Adjusted: Model 1** | | **Adjusted: Model 2** | | **Adjusted: Model 3** | |
| --- | --- | --- | --- | --- | --- | --- | --- | --- |
|  | **Unadjusted OR (95% CI)** | **p value** | **Adjusted OR (95% CI)** | **p value** | **Adjusted OR (95% CI)** | **p value** | **Adjusted OR (95% CI)** | **p value** |
| The whole study population |  |  |  |  |  |  |  |  |
| MACEs^a^ | 1.29 (1.27-1.32) | <0.001 | 1.12 (1.09-1.14) | <0.001 | 1.10 (1.08-1.12) | <0.001 | 1.10 (1.08-1.12) | <0.001 |
| Propensity score-matched population | |  |  |  |  |  |  |  |
| MACEs^a^ | 1.10 (1.07-1.12) | <0.001 | / | / | / | / | / | / |

Model 1: age, sex, body mass index, hypertension, dyslipidemia, current smoking, drinking, history of stroke/TIA, history of carotid stenosis, history of MI, history of atrial fibrillation, history of heart failure, history of PAD, history of liver/renal dysfunction, administration of antiplatelet agent use, administration of anticoagulant therapy and administration of statins, education, insurance and hospital location.

Model 2: adjusted for variables in model 1 plus in-hospital National Institutes of Health Stroke Scale score.

Model 3: adjusted for variables in model 1 plus imputed in-hospital National Institutes of Health Stroke Scale score.

^a^ MACEs were defined as a combination of ischemic stroke, hemorrhagic stroke, and myocardial infarction.

# Table S5 Characteristics of AIS patients with and without diabetes/possible diabetes after propensity-score matching

|  | **Total**  **(N=442,862)** | **Diabetes/possible diabetes**  **(N=221,431)** | **No diabetes**  **(N=221,431)** | **ASD (%)** |
| --- | --- | --- | --- | --- |
| Age, mean (SD), years | 66.1 (11.8) | 66.1 (11.2) | 66.1 (12.4) | 0.0 |
| Women, n (%) | 182,033 (41.1) | 90,479 (40.9) | 91,554 (41.3) | 0.8 |
| BMI, mean (SD) | 24.3 (4.3) | 24.3 (4.1) | 24.3 (4.6) | 0.0 |
| BMI, n (%) |  |  |  |  |
| <24.0 | 223,022 (50.4) | 109,127 (49.3) | 113,895 (51.4) | 4.2 |
| 24.0≤BMI<28.0 | 174,064 (39.3) | 88,888 (40.1) | 85,176 (38.5) | 3.3 |
| ≥28.0 | 45,776 (10.3) | 23,416 (10.6) | 22,360 (10.1) | 1.6 |
| Education, n (%) |  |  |  |  |
| College | 15,228 (3.4) | 7,636 (3.4) | 7,592 (3.4) | 0.0 |
| High school | 141,823 (32.0) | 71,638 (32.4) | 70,185 (31.7) | 1.5 |
| Below Elementary | 130,790 (29.5) | 64,614 (29.2) | 66,176 (29.9) | 1.5 |
| Unclear | 155,021 (35.0) | 77,543 (35.0) | 77,478 (35.0) | 0.0 |
| Insurance, n (%) |  |  |  |  |
| UEBMI | 150,692 (34.0) | 75,106 (33.9) | 75,586 (34.1) | 0.4 |
| URBMI | 87,881 (19.8) | 43,881 (19.8) | 44,000 (19.9) | 0.3 |
| NRCMS | 160,310 (36.2) | 80,305 (36.3) | 80,005 (36.1) | 0.4 |
| Self-pay | 26,788 (6.0) | 13,488 (6.1) | 13,300 (6.0) | 0.4 |
| Other | 17,191 (3.9) | 8,651 (3.9) | 8,540 (3.9) | 0.0 |
| SBP, mean (SD) | 151.3 (22.7) | 151.5 (22.8) | 151.1 (22.7) | 1.8 |
| DBP, mean (SD) | 87.3 (13.7) | 86.8 (13.5) | 87.8 (13.9) | 7.3 |
| Risk factors |  |  |  |  |
| Hypertension, n (%) | 404,104 (91.2) | 201,906 (91.2) | 202,198 (91.3) | 0.4 |
| Dyslipidemia, n (%) | 82,500 (18.6) | 41,758 (18.9) | 40,742 (18.4) | 1.3 |
| Current smoking, n (%) |  |  |  |  |
| Men | 90,686 (34.8) | 45,852 (35.0) | 44,834 (34.5) | 1.1 |
| Women | 4,543 (2.5) | 2,210 (2.4) | 2,333 (2.5) | 0.6 |
| Drinking, n (%) | 98,031 (22.1) | 49,399 (22.3) | 48,632 (22.0) | 0.7 |
| History of diseases |  |  |  |  |
| Prior stroke/TIA, n (%) | 150,414 (34.0) | 75,347 (34.0) | 75,067 (33.9) | 0.2 |
| Prior MI, n (%) | 9,559 (2.2) | 4,883 (2.2) | 4,676 (2.1) | 0.7 |
| Atrial fibrillation, n (%) | 22,138 (5.0) | 11,282 (5.1) | 10,856 (4.9) | 0.9 |
| Heart failure, n (%) | 5,290 (1.2) | 2,710 (1.2) | 2,580 (1.2) | 0.0 |
| Carotid stenosis, n (%) | 6,782 (1.5) | 3,401 (1.5) | 3,381 (1.5) | 0.0 |
| PAD, n (%) | 9,529 (2.2) | 4,896 (2.2) | 4,633 (2.1) | 0.7 |
| Liver/renal dysfunction, n (%) | 5,574 (1.3) | 2,863 (1.3) | 2,711 (1.2) | 0.9 |
| NIHSS, n (%) |  |  |  |  |
| 0-4 | 282,700 (63.8) | 140,104 (63.3) | 142,596 (64.4) | 2.3 |
| 5-14 | 131,149 (29.6) | 67,111 (30.3) | 64,038 (28.9) | 3.1 |
| ≥15 | 29,013 (6.6) | 14,216 (6.4) | 14,797 (6.7) | 1.2 |
| Hospital location, n (%) |  |  |  |  |
| Eastern | 219,021 (49.5) | 109,293 (49.4) | 109,728 (49.6) | 0.4 |
| Central | 134,766 (30.4) | 67,422 (30.4) | 67,344 (30.4) | 0.0 |
| Western | 89,075 (20.1) | 44,716 (20.2) | 44,359 (20.0) | 0.5 |
| In-hospital treatment |  |  |  |  |
| IV-rtPA administration ≤4.5 h, n (%) | 27,764 (6.4) | 12,544 (5.8) | 15,220 (7.0) | 4.9 |
| Antiplatelet drugs, n (%) | 373,014 (85.9) | 187,533 (86.3) | 185,481 (85.5) | 2.3 |
| Aspirin | 329,829 (100.0) | 165,523 (100.0) | 164,306 (100.0) | 0.0 |
| Clopidogrel | 217,122 (100.0) | 111,297 (100.0) | 105,825 (100.0) | 0.0 |
| Aspirin + Clopidogrel | 176,520 (40.6) | 90,533 (41.7) | 85,987 (39.6) | 4.3 |
| Anticoagulant, n (%) | 23,758 (5.5) | 11,805 (5.4) | 11,953 (5.5) | 0.4 |
| unfractionated heparin | 2,099 (0.6) | 1,043 (0.5) | 1,056 (0.6) | 1.4 |
| low molecular weight heparin | 16,894 (4.5) | 8,486 (4.5) | 8,408 (4.5) | 0.0 |
| Warfarin | 4,762 (1.3) | 2,233 (1.2) | 2,529 (1.3) | 0.9 |
| Other anticoagulants | 2,412 (0.6) | 1,184 (0.5) | 1,228 (0.6) | 1.4 |
| Statins, n (%) | 35,794 (8.1) | 18,147 (8.2) | 17,647 (8.0) | 0.7 |

AIS, acute ischemic stroke; ASD, absolute standard difference; BMI, body mass index; UEBMI, urban employee basic medical insurance; URBMI, urban resident basic medical insurance; NRCMS, new rural cooperative medical scheme; SBP, systolic blood pressure; DBP, diastolic blood pressure; TIA, transient ischemic attack; MI, myocardial infarction; PAD, peripheral artery disease; NIHSS, national institutes of health stroke scale; IV-rtPA, intravenous recombinant tissue plasminogen activator

# Table S6 Association between diabetes/possible diabetes and in-hospital outcomes in AIS patients after propensity-score matching

| **Propensity-score matched population** | **Unadjusted OR (95% CI)** | **p value** |
| --- | --- | --- |
| All-cause mortality | 1.26 (1.17-1.35) | <0.001 |
| MACE | 1.07 (1.05-1.10) | <0.001 |
| Recurrent ischemic stroke | 1.12 (1.10-1.15) | <0.001 |
| Recurrent hemorrhagic stroke | 1.00 (0.94-1.06) | 0.964 |
| TIA | 0.92 (0.86-0.99) | 0.021 |
| MI | 1.08 (0.99-1.17) | 0.104 |

AIS, acute ischemic stroke; OR, odds ratio; CI, confidence interval; MACEs: major adverse cardiovascular events; TIA: transient ischemic attack; MI: myocardial infarction

1,006,798 patients in CSCA program from 1,476 hospitals

Patients with acute ischemic stroke included in this study (N=838,229)

Excluding TIA patients (N=64,929)

Excluding hemorrhagic stroke patients (N=85,705)

Excluding subarachnoid hemorrhage patients (N=11,241)

Excluding diagnosed as "undetermined" (N=6,694)

Evaluating prevalence of diabetes (N=838,229)

Evaluating the association between diabetes and in-hospital outcomes (N=824,600)

Evaluating the association between diabetes and in-hospital outcomes after PSM (N=442,862)

Patients with missing data for in-hospital NIHSS score (N=165,993)

Unmatched patients (N=215,745)

PSM

Patients with missing data for multivariable logistic regression (N=13,629)

# **Fig. S1 Flow chart of study**

CSCA: Chinese Stroke Center Alliance, TIA: transient ischemic stroke, PSM: propensity score matching, NIHSS: National Institute of Health Stroke Score.





# Fig. S2 Love plot for absolute standardized differences before and after propensity score matching

BMI, body mass index; MI, myocardial infarction; NIHSS, National Institutes of Health Stroke Scale; NRCMS, new rural cooperative medical scheme; PAD, peripheral artery disease; PSM, propensity score matching; SBP, systolic blood pressure; TIA, transient ischemic attack; UEBMI, urban employee basic medical insurance; URBMI, urban resident basic medical insurance.

**REFERENCE**

1. Zhou B-F (2002) Predictive values of body mass index and waist circumference for risk factors of certain related diseases in Chinese adults--study on optimal cut-off points of body mass index and waist circumference in Chinese adults. Biomedical and environmental sciences: BES 15:83-96.
